# Hysteroscopy as An Investigational Operative Procedure in Primary and Secondary Infertility: A Systematic Review

**DOI:** 10.22074/IJFS.2020.134704

**Published:** 2021-03-11

**Authors:** Fortunato Genovese, Federica Di Guardo, Morena Maria Monteleone, Valentina D’Urso, Francesco Maria Colaleo, Vito Leanza, Marco Palumbo

**Affiliations:** Department of General Surgery and Medical Surgical Specialties, University of Catania, Viale Carlo Azeglio Ciampi, Catania, Italy

**Keywords:** Hysteroscopy, Infertility, Pregnancy Rate, Primary Infertility

## Abstract

**Background:**

The aim of this study is to review current indications to diagnostic and/or operative hysteroscopy in primary and secondary infertility, as well as to determine its efficacy in improving fertility.

**Materials and Methods:**

We gathered available evidence about the role of hysteroscopy in the management of vari- ous infertility conditions. Literature from 2000 to 2020 that pertained to this topic were retrieved and appropriately selected.

**Results:**

Hysteroscopy does not appear as a first line diagnostic procedure for every clinical scenario. However, its di- agnostic sensitivity and specificity in assessing intrauterine pathology is superior to all other non-invasive techniques, such as saline infusion/gel instillation sonography (SIS/GIS), transvaginal sonography (TVS) and hysterosalpingog- raphy (HSG). Hysteroscopy allows not only a satisfactory evaluation of the uterine cavity but also, the eventual treat- ment of endocavitary pathologies that may affect fertility both in spontaneous and assisted reproductive technology (ART) cycles.

**Conclusion:**

Hysteroscopy, due to its diagnostic and therapeutic potential, should be regarded as a necessary step in infertility management. However, in case of suspected uterine malformation, hysteroscopy should be integrated with other tests [three-dimensional (3D) ultrasound or magnetic resonance imaging (MRI)] for diagnostic confirmation.

## Introduction

During the last decade, infertility has had an increasing impact onthe health of Western countries’ populations. The most accepted definition of infertility is failure to conceive after 12 months or more of regular unprotected sexual intercourses (1).

During the last 20 years, multiple factors have been addressed as causes of reduced spontaneous conception, among which intrauterine pathologies might play a crucial role. According to this, several treatments have been proposed to overcome infertility due to the presence of intrauterine affections. In this context, hysteroscopy is currently considered the gold standard for both assessment and management of intrauterine factors. Indeed, it allows a more precise diagnosis of endometrial abnormalities compared to non-invasive techniques such as transvaginal sonography (TVS), hysterosalpingography (HSG) and sonohysterography; above all, it allows for simultaneous treatment of an intrauterine pathology (2).

The present study is a systematic review on the efficacy of diagnostic and/or operative hysteroscopy in improving reproductive outcomes for specific conditions in infertile women.

## Materials and Methods

We systematically reviewed the literature from 2000 to 2020 by searching in PubMed, Embase, and the Cochrane Libraryby using the following keywords: infertility, hysteroscopy, pregnancy rate (PR), miscarriages, live birth rates (LBR), uterine malformations and metroplasty. In general, randomised controlled trials (RCT) were selected; if they were not available on a specific subject, less relevant studies were chosen. The patients included in this review were infertile women with or without endometrial abnormalities who sought spontaneous conception or required* in vitro* fertilization/ intracytoplasmic sperm injection (IVF/ICSI). The type of intervention analysed is diagnostic and/or operative hysteroscopy performed during the infertility evaluation and/or prior IVF/ICSI compared to no hysteroscopy in similar groups of patients. 

### Study population

We divided the studied population according to indication and efficacy of hysteroscopy in improving reproductive outcomes. As result, we obtained the following four groups: group A: initial work-up of asymptomatic patients with negative ultrasound findings; group B: women with endometrial abnormalities at the TVS with or without abnormal uterine bleeding (AUB); group C: patients with genital tract malformations and/or recurrent abortions; and group D: women with negative ultrasound findings who required assisted reproductive technology (ART), IVF or ICSI.

### Main outcomes

The primary outcome was clinical PR (CPR), which was defined by at least TVS visualization of the gestational sac. The secondary outcome was miscarriage rate (MR), which was defined as pregnancy loss before 20 weeks of gestation.

## Results

**Fig.1:**
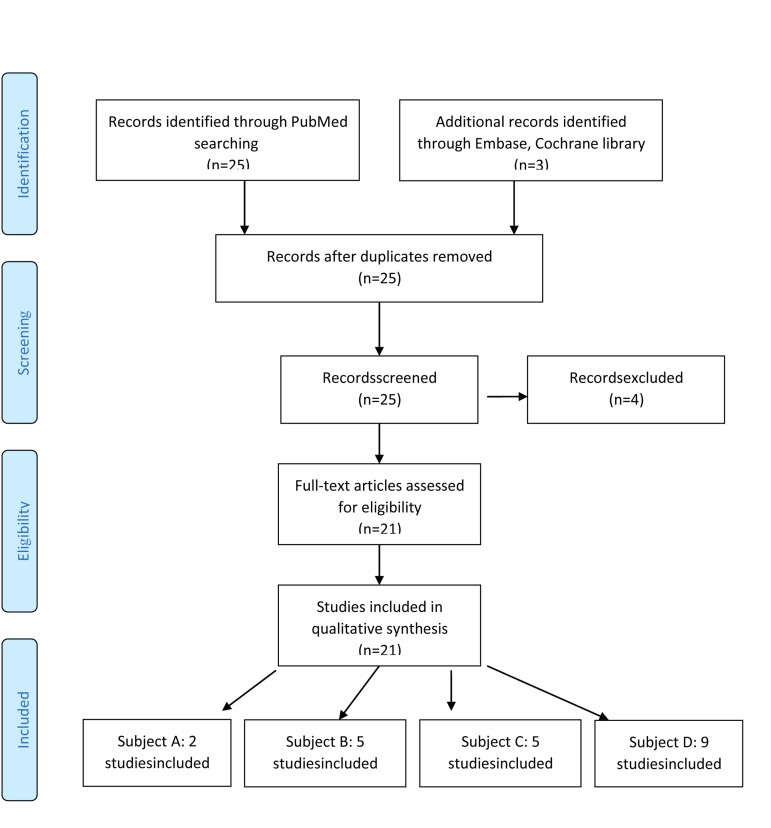
Prisma flow chart (3).

Atotal of 28 records were considered in the study selection process. After removing three duplicates and excluding four studies due to incomplete outcomes, 21 full-text articles wereconsidered suitable for the systematic review (Fig.1).

Subdivisions of the selected articles according to groups A, B, C or D, and their results, are described as follows. Only two studies, one retrospective and one prospective, were included in group A, which showed that a consistent percentage of women (79 and 33%, respectively) had hysteroscopic abnormalities (Table 1). Group B comprised four studies- two prospective, one RCT, and one retrospective cross-sectional. The outcomes reported that 65% of patients achieved pregnancy after hysteroscopy with only one intrauterine insemination (IUI), and the PR was significantly higher after hysteroscopic removal of submucous myomas. Malignancy or atypia did not occur in the subsequent 12 months of followup after the hysteroscopy, and the uterine cavity was restored in 93.6% of women, respectively (Table 2). Group C had five studies, three prospective and two retrospectives. The results revealed significantly higher PR and LBR in patients who underwent hysteroscopic treatment of uterine septum (Table 3). A total of nine RCT studies were assigned to group D. This group had significantly higher PR, CPR, LBR and implantation rate (IR) in the selected categories of patients who underwent hysteroscopy before ICSI (Table 4). Tables 1-4 display the aforementioned groups in extensive details. 

**Table 1:** Characteristics of the two studies included in group A


Author	Country, year	Study design	Main inclusion criteria	Intervention	Control	Results

Di Muzio et al. (4)	Italy, 2016	Retrospective	92 nulliparous patients with unexplained infertility (absence of uterine lesions at TVS and HSG)	All patients underwent diagnostic and operative hysteroscopies	No	PR: 85% (79% of patients had endometrial lesions)
Elbareg et al. (5)	Libya, 2014	Prospective	200 infertile women in whom standard infertility investigations revealed no abnormalities	All women underwent diagnostic and operative hysteroscopies	No	PR: 46%(33% of women had hysteroscopic abnormalities)


TVS; Transvaginal sonography, HSG; Hysterosalpingography, and PR; Pregnancy rate.

**Table 2:** Characteristics of the five studies included in group B


Author	Country, year	Study design	Main inclusion criteria	Intervention	Control	Results

Perez-Medina et al. (6)	Spain, 2005	RCT	204 infertile women with sonographic diagnosis of EP and candidates for IUI	n=101Patients underwent hysteroscopic polypectomy	n=103Patients underwent diagnostic hysteroscopy and polyp biopsy	PR: 63.4% vs. 28.2% (P<0.001)PR (after four IUI cycles) 51.4% vs. 25.4% (P<0.001) (Pregnancies in the study group were obtained before the first IUI in 65% of cases)
Casini et al. (7)	Italy, 2006	RCT	181 infertile women with uterine fibroids	n=92Patients underwent laparotomy or hysteroscopic myomectomy(30 SM; 23 IM; 17 IM-SS and 22 SM-IM)	n=89Patients did not undergo surgical treatment:(22 SM; 22 IM; 11 SS; 14 IM-SS and 20 with IM-SM)	PR (SM): 43.3% vs. 27.2%(P<0.05)PR (IM): 56.5% vs. 41%(P: NS)PR (SM-IM): 36.4% vs. 15%(P<0.05)PR (IM-SS): 35.5% vs. 21.4%(P: NS)
Mazzon et al. (8)	Italy, 2010	Prospective study	6 young nulliparous patients with stage IA endometrial cancer	All patients underwent hysteroscopic resection of the tumour followed by hormone therapy (megestrol acetate,160 mg/day, for 6 months)	No	PR: 66%(no atypia or malignancy after 12 months follow-up)
Chen et al. (9)	China, 2017	Retrospective cross-sectional study	350 infertile women with mild, moderate, and severe IUAs	All patients underwent hysteroscopic adhesiolysis(The reproductive outcomes of 332 cases, 93%, were followed for 27 ± 9 months)	No	PR: 48.2%(60.7% in mild IUAs, 53.4% in moderate, 25% in severe cases)MR: 9.4% LBR: 85.6% Uterine cavity was restored in 93.6% of patients)


EP; Endometrial polyp, SM; Submucosal fibroid, IM; Intramural fibroid, SS; Subserosal fibroid, SM-IM; Submucosal-intramural fibroid, IM-SS; Intramural-subserosal fibroid, IUA; Intrauterine adhesion, IUI; Intrauterine insemination, MR; Miscarriage rate, LBR; Live birth rate, CR; Conception rate, CS; Caesarean section, PAUB; Postmenstrual uterine bleeding, PR; Pregnancy rate, and RCT; Randomised controlled trial.

**Table 3:** Characteristics of the five studiesincluded in group C


Author	Country, year	Study design	Main inclusion criteria	Intervention	Control	Results

Mollo et al. (10)	Italy, 2008	Prospective controlled trial	176 infertile women	n=44Patients withseptate uterus and underwent hysteroscopic metroplasty	n=132Patients with unexplained infertility, managed expectantly	PR: 38.6% vs. 20.4% LBR: 34.1% vs. 18.9%
Tonguc et al. (11)	Turkey, 2010	Retrospective study	127 infertile women with uterine septum	n=102Patients underwent hysteroscopic metroplasty	n=25Patients did not undergo metroplasty	PR: 43.1% vs. 20% (P=0.03)MR: 11.4% vs. 60% (P=0.02)LBR: 35.3% vs. 8% (P=0.008)
Pacheco et al. (12)	Spain, 2019	Prospective cohort study	63 nulliparous infertile womenwith primary T-shaped uterus	All women underwent hysteroscopic metroplasty (Only 60 patients tried to conceive after metroplasty)	No	PR:83.3% LBR:63.3%
Ban-Frangež et al. (13)	Slovenia, 2008	Retrospective matched control study	380 women conceived following IVF/ICSI	n=106Patients underwent hysteroscopic resection ofa small or large septum	n=274 Patients did not undergo surgery because they did not have any uterine malformation	MR (small septum): 30.6% vs. 20.4%(P: NS)MR (large septum): 28.1% vs. 19.3%(P: NS)
Bakas et al. (14)	Greece, 2012	Prospective observational	68 infertile women with septate uterus (12 months follow-up)	All patients underwent hysteroscopic metroplasty	No	CPR: 44%LBR: 36.8%MR: 16.6%


CPR; Clinical pregnancy rate, MR; Miscarriage rate, AR; Abortion rate, LBR; Live birth rate, PR; Pregnancy rate, IVF;* In vitro* fertilization, ICSI; Intracytoplasmic sperm injection, and NS; Not significant.

**Table 4:** Characteristics of the nine studies included in group D


Author	Country, year	Study design	Main inclusion criteria	Intervention	Control	Results

Raju et al. (15)	India, 2006	Prospective RCT	520 women undergoing IVF programme	n=255Had office hysteroscopy-Group A (n=160) hadnormalhysteroscopic findings-Group B(n=95) had abnormal office hysteroscopyfindings that were corrected	n=265Without office hysteroscopy	CPR 44.4% (A) 39.5% (B) vs. 26.2% (P<0.05)
Elsetohy et al. (16)	Egypt, 2014	RCT	193 infertile women with no abnormality detected in TVS undergoing ICSI	n= 97 Hysteroscopic examination before ICSI	n=96ICSI without hysteroscopy	PR: 70.1% vs. 45.8% (P=0.001)
Smit et al. (17)	Netherlands, 2016	Multicentre RCT	742 infertile women scheduled to start IVF or ICSI treatment, with normal TVS	n=369 Hysteroscopy prior to IVF(355 completed 18 months of follow-up)	n=373IVF without hysteroscopic examination(353 completed 18 months of follow-up)	OP: 57%vs. 54% (P=0.41)
Aghahosseini et al. (18)	Iran,2012	RCT	353 women undergoing ICSI withtwo or more implantation failuresand:- no uterine cavity abnormalities- normal HSG - age <38 years.	n=142 Hysteroscopy prior to ART	n=211 Immediate ICSI without prior hysteroscopy	CPR: 50.7% vs. 30.3% Delivery rates was 35.5% in the hysteroscopy group and 21.1% in the control group, respectively
El-Toukhy et al. (19)	UK, Italy, Belgium, Czech Republic, 2016	Multicentre RCT		n=367 IVF cycle with prior hysteroscopy	n=352 IVF cycle without prior hysteroscopy	102 (29%) of women in the hysteroscopy group had a livebirth after IVF compared with 102 (29%) women in the control group (risk ratio 1-0.95% CI 0.79–1.25; P=0.96)
Shawki et al. (20)	Egypt, 2012	RCT	719 infertile women younger than 38 years, with two to four failed IVF cycles and planned a further IVF/ICSI cycle	n=105ICSI after office hysteroscopy	n=110ICSI without office hysteroscopy	IR and CPR were statistically significant between the intervention group and control group
Demirol and Gurgan (21)	Turkey, 2004	RCT	225 infertile women with no uterine factor of infertility,abnormal HSG or TVS, previousintrauterine surgery or contraindication for hysteroscopy	n=210Office hysteroscopic before IVF cycles.Group IIa (n = 154) had normalhysteroscopic findings, and group IIb (n = 56) had abnormal hysteroscopic findings	n=211No office hysteroscopic evaluation before IVF cycles	There was a significant difference in the CPR between patients in the control group and group II a (21.6% and 32.5%, P=0.044, respectively) and control group and group IIb (21.6% and 30.4%, P=0.044, respectively)
El-Nashar and Nasr (22)	Egypt, 2011	RCT	421 patients with primary infertility and two or more failed IVF cycles with no uterine cavity abnormalities and normal HSG	n=62 Hysteroscopy with directed biopsy and correction of any intrauterine abnormalities before ICSI	n = 62ICSI cycle without undergoing a hysteroscopy	CPR: 40.3% vs. 24.2% (P<0.05)
Shohayeb and El-Khayat (23)	Egypt, 2012	Prospective RCT	124 infertile women starting their first ICSI cycle 210 infertile womenwith a history of two or more failed ICSI cycles and withnormal thin endometrium	n=105 Hysteroscopy and endometrial scraping in the cycle preceding the ICSI cycle	n=105 Hysteroscopy without endometrial scraping	IR: 12% vs. 7% (P=0.015). CPR: 32% vs. 18 %(P=0.034) BR 28% vs. 14% (P=0.024)


RCT; Randomized controlled trial, PR; Pregnancy rate, TVS; Transvaginal sonography, HSG; Hysterosalpingography, IR; Implantation rate, MR; Miscarriage rate, LBR; Live birth rate, OP; Ongoing pregnancy rate, ICSI; Intracytoplasmic sperm injection, IVF;* In vitro* fertilization, ART; Assisted reproductive technology, and CPR; Clinical pregnancy rate.

## Discussion

The exploration of the uterine cavity as a routine procedureduring the initial infertility work-up is still under debate. With regards to our study results, only two studies were included in the systematic review, which analysed the role of hysteroscopy in asymptomatic infertile women. The National Institute for Health and Clinical Excellence (NICE guidelines, 2014) stated that hysteroscopy should not be offered during the initial infertility evaluation; on the other hand, according to the Practice Committee of the American Society for Reproductive Medicine (ASRM), hysteroscopy is a relatively expensive and invasive procedure (2). In contrast, the guidelines of the Italian Society of Gynaecological Endoscopy (SEGI), strictly recommend hysteroscopy as a screening procedure for the infertile couple as part of the primary work-up (24), even if a specific evidence of its usefulness in these cases is lacking. Similarly, the literature currently shows an increasing trend towards hysteroscopic evaluation for women who struggle with unexplained infertility. Moreover, this kind of management could help todetect lesions that were not diagnosed by other tools. Indeed, it mayprovide definitive treatment of endocavitary lesions that could impact women fertility (4).

Conversely, hysteroscopic exam of the uterine cavity is considered mandatory during the primary work-up of infertile couples in presence endometrial abnormalities detected at TVS, accompanied or notby bleeding. In this context, the most common endometrial pathologies observed by hysteroscopyare endometrial polyps and submucous fibroids. In general, their treatment by operative hysteroscopy improves PR and reproductive outcomes. Endometrial polyps are thought to interfere with uterine receptivity and embryo implantation, and adversely impact fertility (25). Current evidence supports hysteroscopic resection of endometrial polyps prior to ART in order to improve fertility (6, 25-27). There is a 50% viable PR obtained after polypectomy among subfertile patients (26). In cases with hysteroscopic polypectomy prior to IUI, hysteroscopic removal of polyps showed a significant improvement in clinical PR (27). Submucous fibroids should be also removed in infertile patients, especially if they significantly impact the endometrial line, regardless of the size or the presence of symptoms (27, 28). 

Infertility may be associated with AUB, not only in cases of endometrial polyps and submucous myoma, but also in cases of other endometrial pathologies such as adenomyosis, endometrial hyperplasia and endometrial malignancy. In the latter cases, it is interesting to report that small (<2 cm) intramucous endometrial cancer, well-differentiated, may be removed by hysteroscopy, preserving fertility (29).

Another emerging cause of infertility associated with AUB is isthmocele or uterine scar defects following caesarean section (CS). These may be defined as first, second, or third degree according tothe dimensions. Hysteroscopic treatment of isthmocele is reported to be associated with an increased PR (30).

Intrauterine adhesions (IUA), occasionally associated with Asherman syndrome, are caused by postsurgical or infectious damage to the basalis layer of the endometrium. IUA, sometimes detected on ultrasound as endometrial thickening, may be responsible for infertility and recurrent pregnancy loss (RPL) (31). In this context, hysteroscopy is considered the gold standard for both diagnosis and treatment (32).

Hysteroscopic adhesiolysisis associated with improved fertility as well as reproductive outcomes as reported by Goldenberg et al. (33). Moreover, hysteroscopic evaluation of the uterine cavity is recommended in order to identify eventual congenital uterine abnormalities in patients with RPL (34-36). Indeed, women with a history of recurrent miscarriage or infertility have higher prevalence of congenital uterine anomalies compared with those not having a history of recurrent miscarriage or infertility (37). However, it is important to highlight that, among congenital uterine malformations, septate uterus is the most common structural uterine anomaly associated with the highest incidence of reproductive failure (28). In this context, the Thessaloniki ESHRE/ ESGE consensus on diagnosis of the female genital anomalies has recently established that the combination of gynaecologic examination and two-dimensional (2D)-TVS is recommended as the current standard for the evaluation of asymptomatic women, while threedimensional (3D)-TV is recommended when genital tract anomalies are suspected. Thus, magnetic resonance imaging (MRI) and endoscopic evaluation are also indicated, but only in complex cases or in diagnostic dilemmas (38).

Hysteroscopy, as well as HSG, cannot differentiate septate from bicorporal uterus, due to their inability to assess the contour of the uterus; therefore, both procedures have a limited diagnostic value in the evaluation of genital tract malformations. Conversely, hysteroscopy compared to HSG, may be more useful to investigate the relationship between the cervix (single or double) and the vaginal canal, and analyse the vaginal, the cervical and the uterine intracavitary morphology (39).

When infertility is associated with the presence of a uterine septum, operative hysteroscopy is a valuable tool that offers resolutive management. Bakas et al. (14) have proposed that hysteroscopic metroplasty in patients with septate uterus and unexplained infertility is a method to improve CPR and LBR. Grimbizis et al. (40) reported 6.1% of LBR in untreated women with uterine septum compared with 82% in those who underwent hysteroscopic metroplasty. To date, RCTs with the aim to evaluate the effectiveness and possible complications of hysteroscopic metroplasty have not been published (41). Furthermore, it seems that hysteroscopy with biopsy may be a valid tool in patients with RPL and recurrent implantation failure (RIF) in order to detect chronic endometritis, as reported by Zargar et al. (42).

In ART, the role of hysteroscopy is even more important. In the clinical practice, hysteroscopy is commonly performed before IVF in all patients, including women with normal TVS and/or HSG findings, because a significant percentage may have a misdiagnosed uterine disease that might negatively affect the success of the fertility treatment (43). Hysteroscopy reveals the presence of intrauterine lesions in almost 28% of infertile patients with negative TVS results undergoing ART. This demonstrates that TVS hasa low sensitivity in diagnosis of several intrauterine alterations (44).

Moreover, the RCT by Elsetohy et al. (16), reported that 43.3% of women with negative ultrasounds showed abnormal hysteroscopic findings prior to ICSI. Similarly, an improved IR and CPR, after office hysteroscopy and before undergoing ICSI, was observed, especially in patients whose uterine abnormalities were corrected (20, 45).

El-Toukhy et al. (19) reported significant improvement in PR when hysteroscopy was performed in the cycle before IVF, regardless of intrauterine abnormalities. Possible explanations include possible reliance on irrigation of the cavity with saline, which mechanically removes harmful antiadhesive glycoprotein molecules (46); probing of the cervical canal, which makes the embryotransfer procedure easier (23); and mechanical endometrial injury, which may enhance receptivity by modulating the expressions of gene encoding factors required for implantation (47- 52). Finally, a screening hysteroscopy is recommended prior to ART and highly recommended after two or more failed IVF cycles.

The strength of our study relies on its design. This systematic review included a large sample size of infertile women with or without endometrial abnormalities who sought spontaneous conception or required IVF/ICSI. Despite our robust methodological approach, risk of bias inherent to the nature of the study itself should be taken into consideration when interpreting the results. Larger, prospective randomised studies are warranted to draw firm conclusions.

## Conclusion

Hysteroscopy represents the gold standard for diagnosis and treatment of abnormal uterine findings that are present in approximately 25% of infertile women. These lesions can interfere with spontaneous and assisted reproduction, and may remain undiagnosed with the use of TVS, SIS/GIS or HSG. Although spontaneous or assisted reproductive conception is possible, even in the presence of the small intrauterine abnormalities that represent only 2-3% of infertility causes, their treatment by operative hysteroscopy may help improving the IR and CPR. However, it has to be considered that treatment of intrauterine lesions may not always be synonymous with restoration of fertility. Diagnostic and, if required, operative hysteroscopy prior to ART in infertile women with or without intrauterine abnormalities, may contribute to increase reproductive outcomes.
